# Phosphoproteomic analyses of kidneys of Atlantic salmon infected with *Aeromonas salmonicida*

**DOI:** 10.1038/s41598-019-38890-3

**Published:** 2019-02-14

**Authors:** Peng-fei Liu, Yishuai Du, Lingjie Meng, Xian Li, Dong Yang, Ying Liu

**Affiliations:** 1 Key Laboratory of Experimental Marine Biology, Institute of Oceanology, Chinese Academy of Sciences, Qingdao, 266071 China; 2 Laboratory for Marine Fisheries Science and Food Production Processes, Qingdao National Laboratory for Marine Science and Technology, Qingdao, 266235 China; 3 University of Chinese Academy of Sciences, Beijing, 100039 China; 4 Dalian Ocean University, Dalian, P. R. China; 5 State Key Laboratory of Proteomics, Beijing Proteome Research Center, National Center for Protein Sciences (Beijing), Beijing Institute of Lifeomics, Beijing, 102206 P. R. China

## Abstract

*Aeromonas salmonicida* (*A*. *salmonicida*) is a pathogenic bacterium that causes furunculosis and poses a significant global risk, particularly in economic activities such as Atlantic salmon (Salmo salar) farming. In a previous study, we identified proteins that are significantly upregulated in kidneys of Atlantic salmon challenged with *A*. *salmonicida*. Phosphoproteomic analyses were conducted to further clarify the dynamic changes in protein phosphorylation patterns triggered by bacterial infection. To our knowledge, this is the first study to characterize phosphorylation events in proteins from *A*. *salmonicida*-infected Atlantic salmon. Overall, we identified over 5635 phosphorylation sites in 3112 proteins, and 1502 up-regulated and 77 down-regulated proteins quantified as a 1.5-fold or greater change relative to control levels. Based on the combined data from proteomic and motif analyses, we hypothesize that five prospective novel kinases (VRK3, GAK, HCK, PKCδ and RSK6) with common functions in inflammatory processes and cellular pathways to regulate apoptosis and the cytoskeleton could serve as potential biomarkers against bacterial propagation in fish. Data from STRING-based functional network analyses indicate that fga is the most central protein. Our collective findings provide new insights into protein phosphorylation patterns, which may serve as effective indicators of *A*. *salmonicida* infection in Atlantic salmon.

## Introduction

*Aeromonas salmonicida* subspecies salmonicida (*A*. *salmonicida*) is a gram-negative bacterium that triggers a major infectious and systematic disease affecting a wide range of marine and freshwater fish, especially salmonids^1^. The species can cause death in fish at any growth stage, with symptoms of hemorrhagic septicemia within as little as two or three days^2,3^. Previous studies have revealed symptoms, such as exophthalmia, skin hemorrhage, ulcers and necrosis, in muscle and different internal organs, mainly spleen and kidney^4^. As the major hematopoiesis organ in fish, the kidney has been well characterized in terms of immune responsiveness^5^. Despite its global incidence, the pathogenesis of this infectious disease in fish is currently unknown. Clarification of the mechanisms underlying the immune response to the pathogen infection process and identification of disease indicators should provide a good basis for understanding the bacterial infection resistance ability of Atlantic salmon.

Earlier proteomic analyses by our group using iTRAQ revealed 39 significantly regulated proteins between Atlantic salmon infected with *A*. *salmonicida* and healthy fish at different infection stages (7 and 14 days) and varying degrees of infection (low and high)^6^. However, the specific functions of these and other potential unidentified proteins are yet to be established. A number of studies have demonstrated that phosphorylation regulates pathogenesis. Therefore, to further clarify the mechanisms underlying the response of kidneys of Atlantic salmon to *A*. *salmonicida* infection, we selected the phosphoproteome as a tool to quantify and identify phosphoproteins. In recent years, mass spectrometry-based quantitative proteomic approaches have been extensively used to characterize the phosphoproteome^7^. Significant improvements in enrichment methodologies have been reported^8^. Protein phosphorylation represents a key post-translational modification in many cellular events that exerts regulatory effects on a range of essential biological processes, including metabolism, secretion, homeostasis, transcriptional and translational regulation, and cellular signaling^9^. Phosphoproteome analyses provide critical information on intracellular signaling events as well as the common post-translational modification, phosphorylation, without prior knowledge of function or distribution^10^. In this study, we employed mass spectrometry (MS)-based proteomics combined with phosphopeptide enrichment techniques to identify global protein phosphorylation responses in kidney of Atlantic salmon to infection with *A*. *salmonicida*. These collective findings not only facilitate the identification of phosphoproteins and phosphorylation sites of proteins in kidneys of Atlantic salmon activated in response to *A*. *salmonicida* infection, but also provide a further functional level to distinguish valuable biomarkers for disease diagnosis.

## Materials and Methods

### Fish and bacterial strain preparations

*A*. *salmonicida* strains (CGMCC No. 7335) used in our study were isolated as naturally occurring pathogens in Atlantic salmon cultured in Shandong Oriental Ocean Sci-Tech Co. (Yantai, Shandong Province, China). Affected fish displayed symptoms of furunculosis and mortality. Bacteria from glycerol stocks were incubated at 20 °C for 48–72 h and cultured in tryptone soy agar supplemented with 1% NaCl (w/v). Brain heart infusion (BHI) agar containing 1.5% NaCl was performed to verify that >95% of the bacteria were viable. Bacteria were adjusted to a final count of ~10^8^ CFU/mL^6^.

### Procedure of fish challenge and sampling

All fish in this study were handled in strict accordance with China’s legislation on scientific procedures on living animals. The protocol was approved by the ethics committee at the University of Chinese Academy of Science (Beijing, China). Breeding Atlantic salmon (body weight 113 ± 20 g) were obtained from Shandong Oriental Ocean Sci-Tech Co. Fish were transferred to cycle-filtered plastic tanks and reared on commercial dry pellets (42% crude protein and 22% crude lipid, Beijing Han Yeanye Science & Technology CO., LTD, Beijing, China) daily and acclimatized temporarily for two weeks (18 °C). Each tank was supplied with fresh water, and the oxygen concentration and temperature monitored daily. Thirty-six fish judged as healthy based on clinical parameters were randomly selected from the experimental group and divided into three groups (two infected and one control). Fish in the two infected groups were challenged with the bacterial suspension at final doses of 10^7^ CFU/mL and 10^4^ CFU/mL, respectively. Fish were challenged for 1 h in separate tanks. Under the same conditions, 12 fish in the control groups were placed in a bath with phosphate-buffered saline solution passed through a 0.22 μm membrane filter. Following the challenge, fish were transferred to their original tanks. No mortality was observed during the experimental period. Kidneys from four fish in infected and PBS incubation groups were collected at 0, 7 and 14 days.

### Protein extraction and trypsin digestion

Total protein extraction and purification procedures were performed according to previously described methods^6^. Fish kidney samples were initially transferred to a 5 mL centrifuge tube and sonicated three times on ice using a high intensity ultrasonic processor (Scientz, Ningbo, China) in lysis buffer (8 M urea, 2 mM EDTA, 10 mM DTT and 1% Protease Inhibitor Cocktail). The remaining debris was removed by centrifugation at 20,000 g at 4 °C for 10 min and the supernatant transferred to new tubes. The protein concentration was determined with a 2-D Quant kit (GE, Boston, America) according to the manufacturer’s instructions.

Protein solution was reduced with 10 mM dithiothreitol (DTT) for 1 h at 37 °C and alkylated with 20 mM iodoacetamide for 45 min at room temperature in the dark. For trypsin digestion, the protein sample was diluted by adding 100 mM TEAB to urea at a concentration less than 2 M. Finally, trypsin was added at a trypsin-to-protein mass ratio of 1:50 for the first digestion overnight and 1:100 for a second 4 h digestion. Approximately 50 μg protein for each sample was digested with trypsin for subsequent experiments. Acquired peptides were dissolved in solvent A (0.1% FA in 2% ACN) and directly loaded onto a reversed-phase analytical column (Thermo, USA).

### iTRAQ labeling of proteins for proteomic data analysis

The peptide mixture obtained was labeled using the iTRAQ Reagent-8plex Multiplex Kit (AB SCIEX, USA) according to the manufacturer’s protocol. The experimental groups, each including three biological replicates, were respectively labeled as 113, C-7 d, 114, C-14 d, 115, L-I-7 d, 116, H-I-7 d, 117, L-I-14 d and 118, H-I-14 (Control: C, Low-infected group: L-I, High-infected group: H-I). All labeled samples were separated with a gradient of 2–60% acetonitrile in 10 mM ammonium bicarbonate, pH 10, over 80 min into 12 fractions with an equal volumes collected via HPLC (Thermo DINOEX Ultimate 3000 BioRS, USA). Fractionated samples were analyzed via liquid chromatography-electrospray ionization-tandem mass spectrometry (LC-ESI-MS/MS) performed on an AB SCIEX nanoLC-MS/MS (Thermo Q exactive Orbitrap, USA) system. Subsequent procedures were performed according to previous reports^6^.

The original MS/MS file data were submitted to MaxQuant with the integrated Andromeda search engine (v.1.5.2.8) for data analysis. Raw data files were processed and quantified using Maxquant v1.5.7.4 (21) and searched against the Atlantic salmon database in the NCBI website (97,714 items, updated on December 2015) concatenated with the reverse decoy database. Trypsin/P was specified as the cleavage enzyme allowing up to 2 missing cleavages. Mass error was set to 10 ppm for precursor ions and 0.02 Da for fragment ions. Carbamidomethylation on Cys was specified as fixed modification and oxidation on Met. False discovery rate (FDR) thresholds for protein, peptide and modification sites were specified as 1%. Minimum peptide length was set at 7. We set a 1.5-fold change (which reduced the false positive to a better extent than 2.0-fold change) and p-value < 0.05 as the threshold to distinguish significant changes in data.

### Tandem mass tag (TMT) labeling of proteins for phosphoproteomic data analysis

To identify the protein phosphorylation events in kidney of Atlantic salmon, we applied multiplexed TMT and LC/LC-MS/MS approaches to quantify the phosphoproteomes of healthy fish and those infected with *A*. *salmonicida* for 7 d with biological replicates. The three biological replicates were labeled as 126, K-C-1, 127, K-C-2, 128, K-C-3, 129, K-I-2 and 130, K-I-3 (Control: C, High-infected group: I). TMT-6 plex was selected for quantification. Briefly, one unit of TMT reagent (defined as the amount of reagent required to label 100 μg protein) was thawed and reconstituted in 24 μL ACN. Peptide mixtures were incubated for 2 h at room temperature, pooled, desalted and dried via vacuum centrifugation.

The sample was fractionated using high pH reverse-phase HPLC on an Agilent 300 Extend C18 column (5 μm particles, 4.6 mm ID, 250 mm length). The method used was the same as that described in section 2.4. After combining into 8 overall fractions, peptides were dried via vacuum centrifugation.

Peptide mixtures were initially incubated with an IMAC microsphere suspension with vibration. IMAC microspheres with enriched phosphopeptides were collected via centrifugation and the supernatant removed^11^. To eliminate nonspecifically adsorbed peptides, IMAC microspheres were sequentially washed with 50% ACN/6% TFA and 30% ACN/0.1% TFA. To elute enriched phosphopeptides from IMAC microspheres, elution buffer containing 10% NH_4_OH was added and elution performed with vibration. The supernatant containing phosphopeptides was collected and lyophilized for LC-MS/MS analysis.

The resulting MS/MS data on the phosphoproteome were processed using the same search engine as that for proteomic data analysis, except for the processes of phosphorylation at Ser, Thr, Tyr and acetylation at the protein N-terminus, which were specified as variable modifications. The site localization probability set as >0.75.

### Proteome and phosphoproteome analyses of kidneys of atlantic salmon infected with *A*. *salmonicida*

Gene ontology (GO) analysis was performed using STRING version 10.5 (https://string-db.org)^12^. Proteins and phosphoproteins were classified as regulated based on inclusion of at least one regulated phosphosite. The Encyclopedia of Genes and Genomes (KEGG) database was used to identify enriched pathways. For each category, a two-tailed Fisher’s exact test was employed to establish enrichment of differentially expressed against all identified proteins. Correction for multiple hypothesis testing was carried out using standard false discovery rate control methods. GO and pathways with corrected p-values < 0.05 were considered significant. All the pathways identified were classified into hierarchical categories according to the KEGG website.

### Network analysis

Proteins were filtered if they were not sampled in at least two of the replicates. All common protein and phosphoprotein candidates were searched against STRING database version 10.5 for protein-protein interactions. We followed these initial filtering steps with STRING analysis^13^. Only interactions between proteins belonging to the searched dataset were selected, thereby excluding external candidates. STRING defines a metric designated “confidence score” to classify interaction confidence. We fetched all interactions with confidence scores ≥0.7, representing high confidence. The interaction network from STRING was visualized in Cytoscape, a graph-theoretical clustering algorithm.

### Identification of over-represented kinase substrate motifs

The software tool Motif-x was used to analyze the models of sequences constituted with amino acids in specific positions of modify-21-mers (10 residues upstream and downstream of the site) in all protein sequences^14^. All database protein sequences were used as the background parameter, with other parameters set as default. To further determine whether the identified phosphoproteins activate additional kinases, we investigated the influence of amino acids in the immediate vicinity of each phosphorylation site on phosphorylation of kinase substrates using the Group-based Prediction System (GPS)^15^.

## Results

### Selection of fish infected with *A*. *salmonicida* at different doses and times

We sampled fish at two stages, specifically, 7 d and 14 d after infection. The two infection times were selected to determine the potential proteins involved in the early and late stages of infection. Based on LD_50_ (10^4^ CFU/mL), we selected 10^7^ and 10^4^ CFU/mL as high and low infection doses for challenge. Kidney is the major hematopoiesis organ in fish that performs key roles in immune responsiveness and clearing bacteria from blood^16,17^. Accordingly, kidneys of normal and infected fish (both low and high doses) were isolated at 7 d and 14 d for iTRAQ analysis. We only determined the phosphorylation state of proteins in kidney samples challenged with the high bacterial dose (10^7^ CFU/mL) at 7 d, with a view to improving analysis and identifying the molecules involved in the early stages of infection.

### Multiplexed quantitative analysis of the whole proteome and phosphoproteome

**Figure 1:**
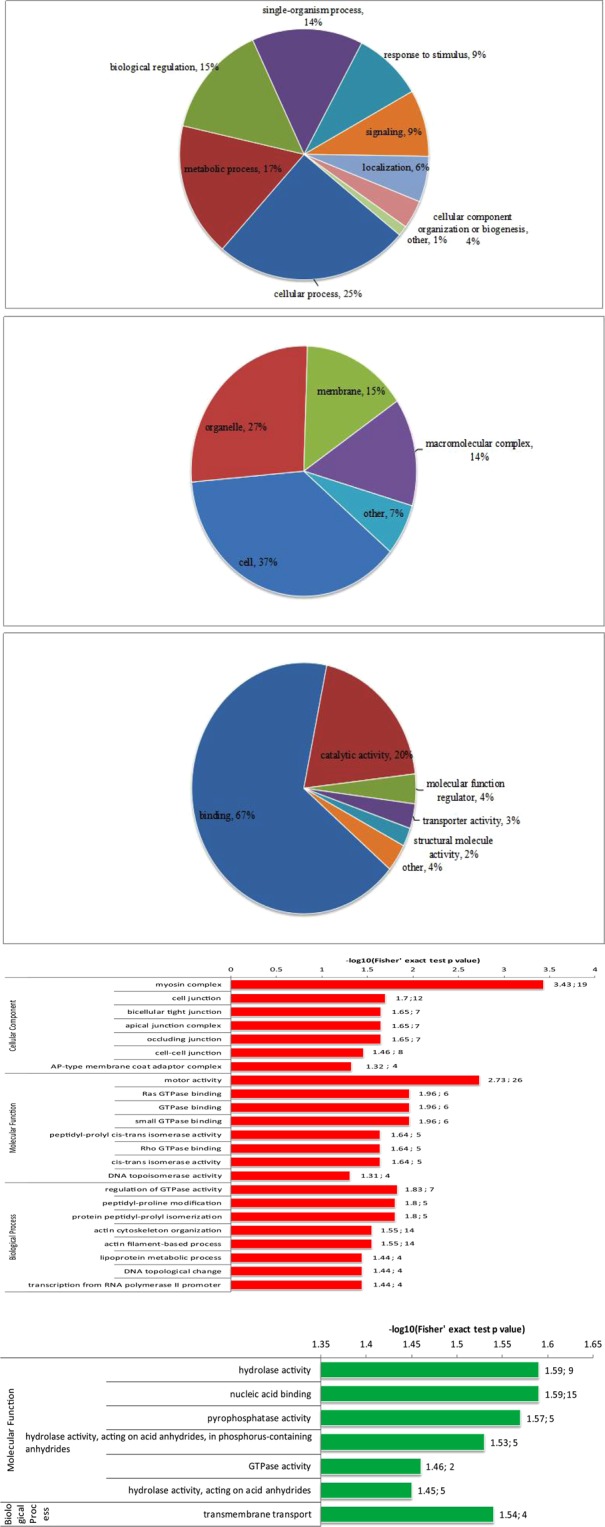
Gene Ontology classification of proteins between high-infected and control groups at 7 d, including (**A**) biological processes, (**B**) cellular components and (**C**) molecular functions. (**D**) GO-based enrichment analysis of up-regulated proteins between high-infected and control groups at 7 d. (**E**) GO-based enrichment analysis of down-regulated proteins between high-infected and control groups at 7 d.

In a previous study, we established a fish model infected with *A*. *salmonicida*^6^. MS-based iTRAQ quantitative proteomics in combination with phosphopeptide enrichment was employed to identify phosphopeptides and phosphoproteins and quantify their expression levels. In total, 5635 phosphorylation sites in 3112 proteins were detected in kidneys of fish, and 1502 and 77 proteins were quantitatively increased and decreased by ≥1.5-fold, respectively (Supplemental Table 1). For proteins possessing multiple phosphorylation sites, GO was used to analyze total phosphorylation in the high-infection group, compared to controls at 7 d. As a result, differentially phosphorylated proteins were sub-categorized into 26 hierarchically structured GO classifications, including 9 biological processes, 5 cellular components and 6 molecular functions (Fig. 1A–C). In particular, “metabolic process” (17%) and “cellular process” (25%) were highly represented in “biological process”, “cell” (37%) and “organelle” (27%) in “cellular component”, and “catalytic activity” (20%) and “binding” (67%) in “molecular function” branches of ontology.

All phosphoproteins involved in biological processes, cellular components and molecular functions were differentially phosphorylated as log10 transformations relative to their average Con expression levels during infection in fish kidney (Fig. 1D,E). Most of the upregulated proteins were associated with myosin complex, motor activity, Ras GTPase binding, GTPase binding, small GTPase binding and regulation of GTPase activity while downregulated proteins were predominantly linked with hydrolase, nucleic acid binding and pyrophosphatase activity.

**Figure 2:**
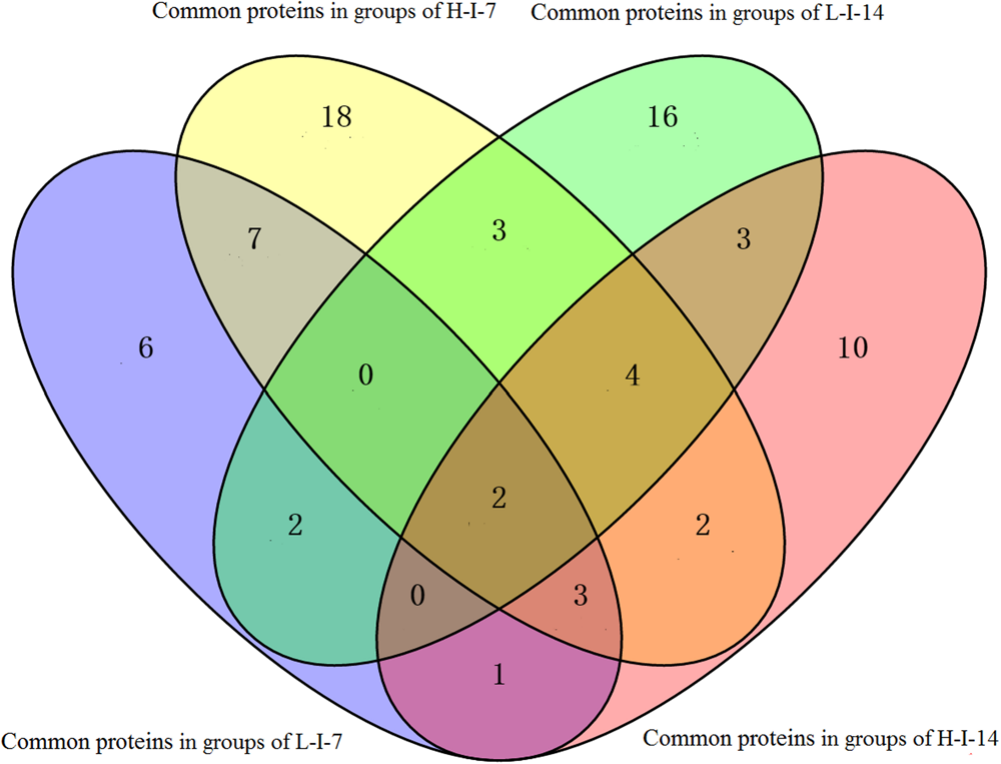
Venn diagram of the numbers of proteins from both whole proteome and phosphoproteome in groups of L-I-7, H-I-7, L-I-14 and H-I-14 significantly expressed proteins at least 1.5 fold with statistical significance (q < 0.05) compared with those in controls respectively.

**Figure 3:**
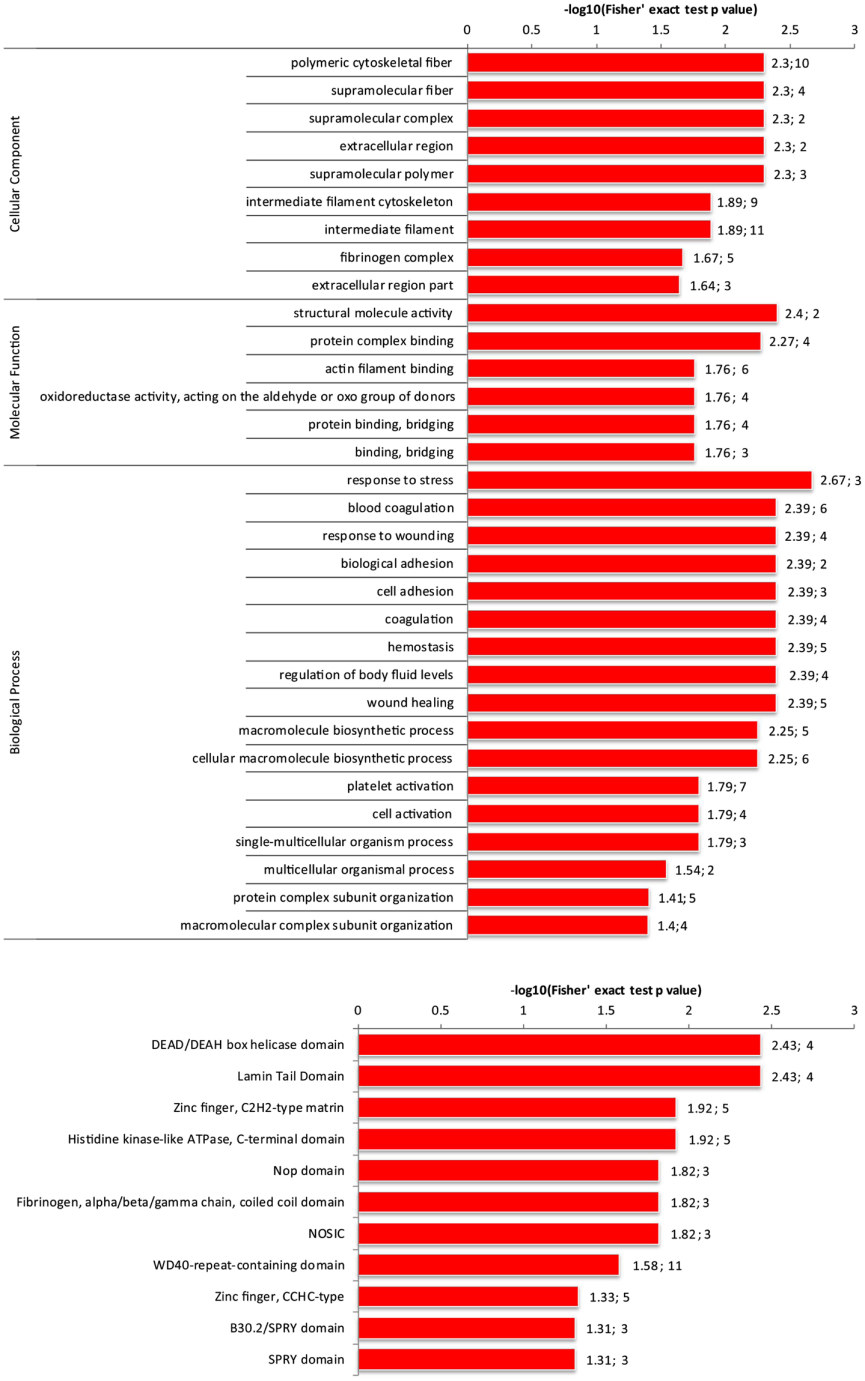
Enrichment of common proteins in both the whole proteome and phosphoproteome. (**A**) Gene Ontology of common proteins; (**B**) Domain enrichment of common proteins.

Samples at 7 and 14 d after *A*. *salmonicida* treatment exhibited alterations in expression in 189 (L-I-7), 244 (H-I-7), 161 (L-I-14) and 135 (H-I-14) proteins by at least 1.5-fold with statistical significance (p < 0.05), compared with the Control group. Interestingly, 21, 39, 30 and 25 proteins were exclusively identified as phosphoproteins in the four treatment groups respectively (Fig. 2). Enrichment analysis further revealed that these proteins are mainly involved in the apoptosis pathway and display characteristic structural features, such as DEAD/DEAH box helicase domain, Lamin Tail Domain, Zinc finger, C_2_H_2_-type matrin, Histidine kinase-like ATPase, C-terminal domain, Nop domain and Fibrinogen, alpha/beta/gamma chain, and coiled coil domain (Fig. 3A,B).

To further determine the differentially phosphorylated and coordinative proteins, we selected significantly altered phosphoproteins with different trends in expression at 7 d (listed in Table 1). Overall, 8 and 11 proteins were identified in the high-dose and low-dose infection groups at 7 d, respectively. Five of the proteins (keratin, type II cytoskeletal cochleal-like, prothrombin-like, membrane-associated progesterone receptor component 1, fibrinogen alpha chain-like, and apolipoprotein A-I precursor) were common between the two groups. Notably, only five (epoxide hydrolase 1, LOW QUALITY PROTEIN: neuroblast differentiation-associated protein AHNAK-like, matrix metalloproteinase-9 precursor, stanniocalcin and lymphocyte-specific protein 1-like) and three (keratin, type I cytoskeletal 18-like, phosphoenolpyruvate carboxykinase, cytosolic [GTP] and hematopoietic cell-specific Lyn substrate 1 isoform X1) proteins were specifically induced in the H-I-7 and L-I-7 treatment groups, respectively, which could potentially serve as indicators of resistance against *A*. *salmonicida* infection.

**Table 1:** List of proteins identified and quantified in kidneys of Atlantic salmon infected with *A*. *salmonicida* from phosphoproteome.

NCBI Accession	Protein description	Protein level	Phosphorylation level	Site	Fold change
**Identified at 7 day of low-concentration infected group**					
NP_001133921.1	phosphoenolpyruvate carboxykinase, cytosolic [GTP] [Salmo salar]	down	up	Ser119	1.965
NP_001134612.1	Apolipoprotein A-I precursor [Salmo salar]	down	up	Ser50	2.567
NP_001139831.1	Membrane-associated progesterone receptor component 1 [Salmo salar]	down	up	Thr63	2.062
XP_013981919.1	PREDICTED: prothrombin-like [Salmo salar]	down	up	Ser319	1.816
XP_014002674.1	PREDICTED: keratin, type II cytoskeletal cochleal-like [Salmo salar]	down	up	Ser273	1.579
XP_014002674.1	PREDICTED: keratin, type II cytoskeletal cochleal-like [Salmo salar]	down	up	Ser31	1.882
XP_014012273.1	PREDICTED: fibrinogen alpha chain-like [Salmo salar]	down	up	Ser443	2.294
XP_014012273.1	PREDICTED: fibrinogen alpha chain-like [Salmo salar]	down	up	Ser457	2.367
XP_014012273.1	PREDICTED: fibrinogen alpha chain-like [Salmo salar]	down	up	Ser433	4.259
XP_014023450.1	PREDICTED: keratin, type I cytoskeletal 18-like [Salmo salar]	down	up	Ser152	1.547
XP_014023450.1	PREDICTED: keratin, type I cytoskeletal 18-like [Salmo salar]	down	up	Ser25	1.875
XP_014063638.1	PREDICTED: hematopoietic cell-specific Lyn substrate 1 isoform X1 [Salmo salar]	down	up	Ser148	1.981
XP_014063638.1	PREDICTED: hematopoietic cell-specific Lyn substrate 1 isoform X1 [Salmo salar]	down	up	Ser11	3.147
NP_001133929.1	Matrix metalloproteinase-9 precursor [Salmo salar]	down	up	Ser27	2.015
NP_001134612.1	Apolipoprotein A-I precursor [Salmo salar]	down	up	Ser50	2.567
NP_001134917.1	Epoxide hydrolase 1 [Salmo salar]	up	down	Ser117	0.642
NP_001139831.1	Membrane-associated progesterone receptor component 1 [Salmo salar]	down	up	Thr63	2.062
XP_013981919.1	PREDICTED: prothrombin-like [Salmo salar]	down	up	Ser319	1.816
XP_013983369.1	PREDICTED: lymphocyte-specific protein 1-like [Salmo salar]	down	up	Ser15	5.749
XP_013993347.1	PREDICTED: stanniocalcin [Salmo salar]	down	up	Ser247	2.08
XP_013993347.1	PREDICTED: stanniocalcin [Salmo salar]	down	up	Ser226	2.584
XP_014002674.1	PREDICTED: keratin, type II cytoskeletal cochleal-like [Salmo salar]	down	up	Ser273	1.579
XP_014002674.1	PREDICTED: keratin, type II cytoskeletal cochleal-like [Salmo salar]	down	up	Ser31	1.882
XP_014002674.1	PREDICTED: keratin, type II cytoskeletal cochleal-like [Salmo salar]	down	up	Ser959	2.429
XP_014012273.1	PREDICTED: fibrinogen alpha chain-like [Salmo salar]	down	up	Ser443	2.294
XP_014012273.1	PREDICTED: fibrinogen alpha chain-like [Salmo salar]	down	up	Ser457	2.367
XP_014012273.1	PREDICTED: fibrinogen alpha chain-like [Salmo salar]	down	up	Ser433	4.259
XP_014054044.1	PREDICTED: LOW QUALITY PROTEIN: neuroblast differentiation-associated protein AHNAK-like [Salmo salar]	down	up	Ser123	1.628
XP_014054044.1	PREDICTED: LOW QUALITY PROTEIN: neuroblast differentiation-associated protein AHNAK-like [Salmo salar]	down	up	Ser2649	1.659
XP_014054044.1	PREDICTED: LOW QUALITY PROTEIN: neuroblast differentiation-associated protein AHNAK-like [Salmo salar]	down	up	Ser2621	1.757
XP_014054044.1	PREDICTED: LOW QUALITY PROTEIN: neuroblast differentiation-associated protein AHNAK-like [Salmo salar]	down	up	Ser2651	1.816
XP_014054044.1	PREDICTED: LOW QUALITY PROTEIN: neuroblast differentiation-associated protein AHNAK-like [Salmo salar]	down	up	Ser130	1.842
XP_014054044.1	PREDICTED: LOW QUALITY PROTEIN: neuroblast differentiation-associated protein AHNAK-like [Salmo salar]	down	up	Ser1228	1.876
XP_014054044.1	PREDICTED: LOW QUALITY PROTEIN: neuroblast differentiation-associated protein AHNAK-like [Salmo salar]	down	up	Ser2678	2.774
XP_014054044.1	PREDICTED: LOW QUALITY PROTEIN: neuroblast differentiation-associated protein AHNAK-like [Salmo salar]	down	up	Ser1437	2.79
XP_014054044.1	PREDICTED: LOW QUALITY PROTEIN: neuroblast differentiation-associated protein AHNAK-like [Salmo salar]	down	up	Ser704	3.184
XP_014054044.1	PREDICTED: LOW QUALITY PROTEIN: neuroblast differentiation-associated protein AHNAK-like [Salmo salar]	down	up	Ser514	3.47
XP_014054044.1	PREDICTED: LOW QUALITY PROTEIN: neuroblast differentiation-associated protein AHNAK-like [Salmo salar]	down	up	Ser2733	3.626
XP_014054044.1	PREDICTED: LOW QUALITY PROTEIN: neuroblast differentiation-associated protein AHNAK-like [Salmo salar]	down	up	Ser598	4.952
XP_014054044.1	PREDICTED: LOW QUALITY PROTEIN: neuroblast differentiation-associated protein AHNAK-like [Salmo salar]	down	up	Ser2739	5.71
XP_014054044.1	PREDICTED: LOW QUALITY PROTEIN: neuroblast differentiation-associated protein AHNAK-like [Salmo salar]	down	up	Ser1554	9.949
XP_014068765.1	PREDICTED: LOW QUALITY PROTEIN: neuroblast differentiation-associated protein AHNAK-like [Salmo salar]	down	up	Ser4497	1.677
XP_014068765.1	PREDICTED: LOW QUALITY PROTEIN: neuroblast differentiation-associated protein AHNAK-like [Salmo salar]	down	up	Ser356	1.957
XP_014068765.1	PREDICTED: LOW QUALITY PROTEIN: neuroblast differentiation-associated protein AHNAK-like [Salmo salar]	down	up	Ser4533	1.984

Numbers on the bar chart represent−log10(*p*) and GO-terms level.

### Motif analysis and regulation of protein kinases and phosphatases

**Figure 4:**
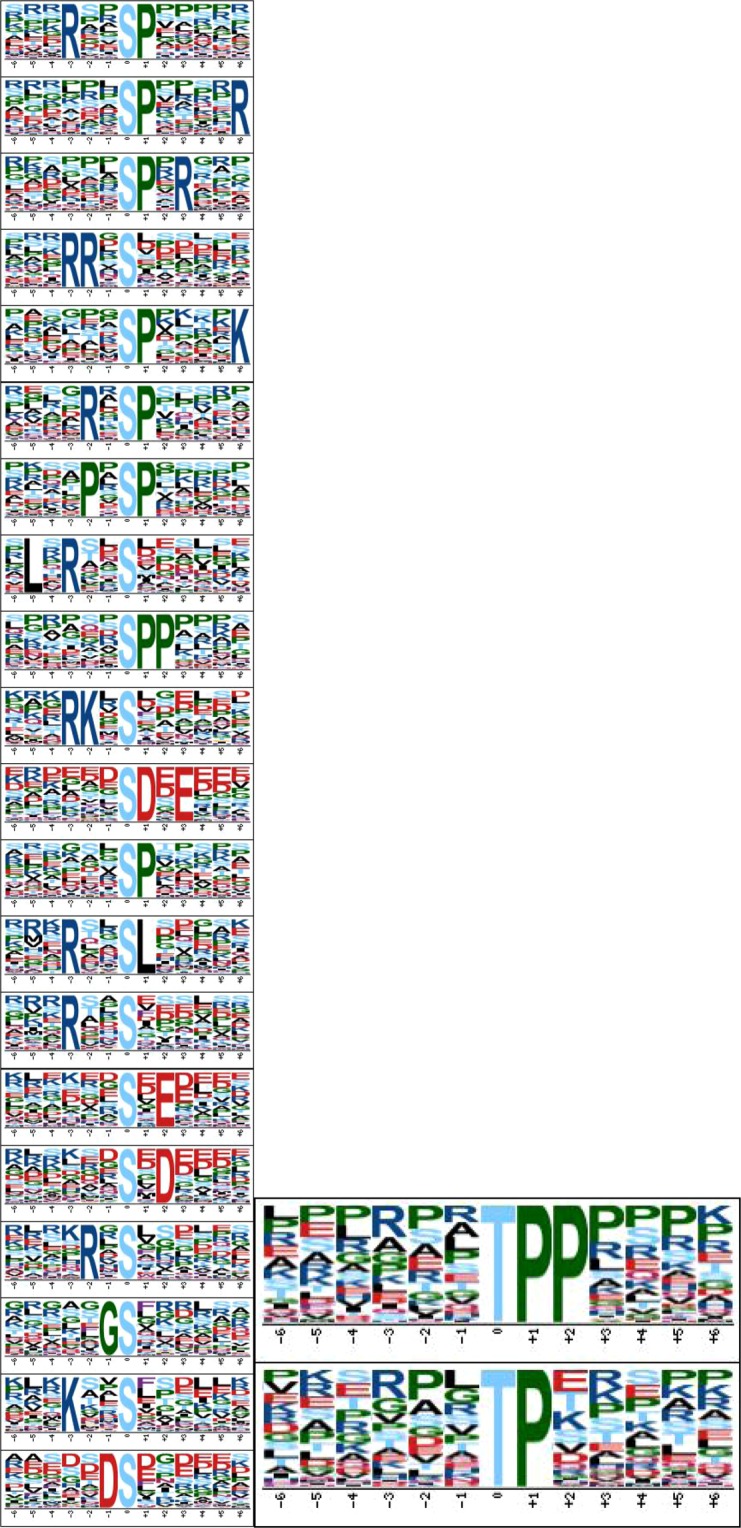
Search for putative kinase substrate motifs within up- (**A**) and downregulated (**B**) *A*. *salmonicida* responsive phosphorylation sites. Representative phosphorylation motifs identified from proteins at Ser (F) and Tyr (E) and centered on phosphorylated serine are shown for each top-scoring motif.

Application of motif-x to determine the amino acid sequence patterns resulted in the identification of 22 highly enriched motifs including Ser and Tyr among all the phosphoproteins (Fig. 4A,B). Proteins were assigned as typical kinases based on an online search of the kinase database (http://www.kinase.com/human/kinome/), mainly including members of STE (17), CAMK (20), AGC (19), CK1(4), CMGC (13), TK (22), TKL (15), RGC (1) and other families (Supplemental Table 2). Additionally, the motif-x tool revealed significant overrepresentation of eight motifs (SxDx, xGSx, RxSx, KxxS, RxxS, RxxSL, RKxS and LXRxxS) among the upregulated *A*. *salmonicida*-responsive phosphorylation sites. These clustered proteins are presented in Supplemental Fig. 1. Combined with the protein data presented in Table 1, five proteins, specifically, inactive serine/threonine-protein kinase VRK3-like, cyclin-G-associated kinase isoform X2, tyrosine-protein kinase HCK isoform X1, protein kinase C delta type-like and ribosomal protein S6 kinase alpha-3-like, were identified as potential biomarkers matched three motifs including SxDx, RxSx and KxxS respectively were shown in Table 2. SxDx corresponds to the consensus motif of inactive serine/threonine-protein kinase, VRK3-like, with Ser118 and Ser151 phosphorylation sites, and cyclin-G-associated kinase isoform, X2, with a Ser1049 phosphorylation site and RxSx to the consensus motif for tyrosine-protein kinase HCK isoform X1 with a Ser236 phosphorylation site. The activities of protein kinase C delta type-like with Ser658, Ser297 and Ser344 phosphorylation sites and ribosomal protein S6 kinase alpha-3-like with Ser363 and Ser221 phosphorylation sites are regulated by the KxxS motif. The phosphorylation status of these kinase proteins may serve as an effective biomarker of *A*. *salmonicida* infection in Atlantic salmon.

**Table 2:** Kinases identified in *A*.

NCBI Accession	Protein description	Kinase Name	Kinase Group	Kinase Family	Site	Motif
XP_014031257.1	PREDICTED: inactive serine/threonine-protein kinase VRK3-like [Salmo salar]	VRK3	CK1	VRK	Ser118	……S.D….
XP_014031257.1	PREDICTED: inactive serine/threonine-protein kinase VRK3-like [Salmo salar]	VRK3	CK1	VRK	Ser151	……S.D….
XP_014028116.1	PREDICTED: cyclin-G-associated kinase isoform X2 [Salmo salar]	GAK	Other	NAK	Ser1049	……S.D….
XP_013989897.1	PREDICTED: tyrosine-protein kinase HCK isoform X1 [Salmo salar]	HCK	TK	Src	Ser236	….R.S……
XP_014022605.1	PREDICTED: protein kinase C delta type-like [Salmo salar]	PKCd	AGC	PKC	Ser658	…K..S……
XP_014022605.1	PREDICTED: protein kinase C delta type-like [Salmo salar]	PKCd	AGC	PKC	Ser297	…K..S……
XP_014022605.1	PREDICTED: protein kinase C delta type-like [Salmo salar]	PKCd	AGC	PKC	Ser344	…K..S……
XP_013984775.1	PREDICTED: ribosomal protein S6 kinase alpha-3-like [Salmo salar]	RSK2	AGC	RSK	Ser363	…K..S……
XP_013984775.1	PREDICTED: ribosomal protein S6 kinase alpha-3-like [Salmo salar]	RSK2	AGC	RSK	Ser221	…K..S……

*Salmonicida* infection in kidney of Atlantic Salmon by phosphoproteomics.

### Network of phosphoproteins

**Figure 5:**
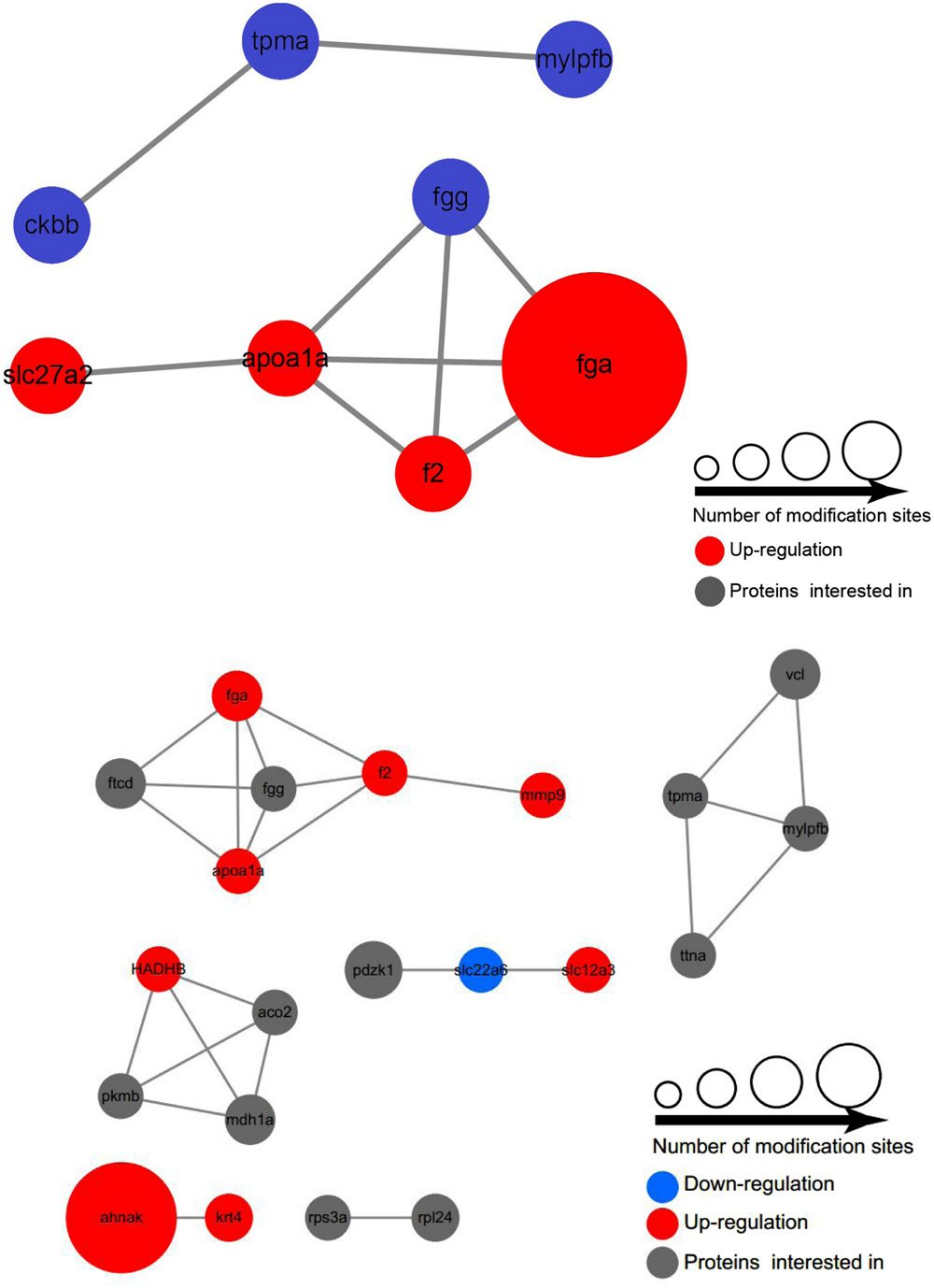
Interaction networks of *A*. *salmonicida*-responsive phosphoproteins. Functional and physical interactions among genes with *A*. *salmonicida*-responsive phosphosites were extracted from the STRING database (version 10.5). Upregulated proteins are represented by red nodes, downregulated proteins by blue nodes, and overlap between up- and downregulated sites by gray nodes. All genes are specified in Supplemental Table 1 (Supporting Information). (**A**) Proteins further selected in low-infected groups at 7 d. (**B**) Proteins further selected in high-infected groups at 7 d.

Using the STRING database, interactions among 290 phosphoproteins from both the proteome and phosphoproteome were identified, including 111 increased and 2 decreased at the phosphorylation level, with filter criteria of log2 ratios > 2 representing increased proteins and log2 ratios < 2 representing decreased proteins (Supplemental Fig. 2). We had filtered different significantly up or down regulated phosphoproteins with specific motifs in previous analyses. Details of these proteins are summarized in Supplemental Table 2 (Supporting Information). Combined with previous findings, significantly up or down regulated phosphoproteins from both low-infected and high-infected groups, compared to controls, at 7 d were selected (Fig. 5A,B), including fibrinogen alpha chain (fga), prothrombin-like (f2), apolipoprotein A-I precursor (apoa1a), ribosomal protein S6 kinase alpha-3-like (rps6ka3a), hematopoietic cell-specific Lyn substrate 1 isoform X1 (hcls1), membrane-associated progesterone receptor component 1 (pgrmc1) and trifunctional enzyme subunit beta, mitochondrial (HADHB). Among the proteins identified, only fga clustered within the network acted as a central rod.

## Discussion

In the current investigation, quantitative proteomics was applied to analyze the phosphoproteome in Atlantic salmon infected with *A*. *salmonicida*. To our knowledge, this is the first study to characterize the phosphorylation events in infected Atlantic salmon. Based on proteomic data obtained from our previous experiments^6^ in combination with phosphoproteomic data, we identified four proteins (epoxide hydrolase 1, neuroblast differentiation-associated protein AHNAK-like, matrix metalloproteinase-9 precursor, stanniocalcin and lymphocyte-specific protein 1-like) that were significantly altered following infection.

The soluble epoxide hydrolase (sEH), a ubiquitous enzyme in vertebrates that transforms epoxides to their corresponding diols implicated in the inflammatory cascade, plays broad biological roles by virtue of its activity against lipid epoxide substrates and localization in not only liver but also kidneys and vascular tissue^18–21^. sEH is a bifunctional enzyme with C-terminal hydrolase activity and N-terminal phosphatase activity^22^. Interestingly, Skugor *et al*. (2009) observed elevated expression of biotransformation genes, such as ephx1, in Atlantic salmon with high resistance against *A*. *salmonicida* infection, highlighting a possible role in tissue protection and toxic metabolite clearance^23^.

Matrix metalloproteinase-9 (MMP-9), one of the members of the MMP family, functions in normal and neoplastic invasive processes, cleaves numerous structural components and participates in inflammatory pathways^24,25^. MMP-9 cleaves a diverse range of substrates, such as extracellular matrix (ECM) proteins, ligands and receptors, and is mainly produced by inflammatory cells, including macrophages, leukocytes and monocytes. The protein plays important roles in embryogenesis, innate immune defense and apoptosis^26,27^. In earlier studies, expression of MMP-9 could be induced by bacterial pathogens in fish, suggesting its involvement in fish immune responses^28–30^. It is believed that these two proteins contribute to defense against *A*. *salmonicida* infection in Atlantic salmon, but further research is required to validate this hypothesis. Few studies have characterized the roles of neuroblast differentiation-associated protein, AHNAK-like and stanniocalcin and lymphocyte-specific protein 1-like during bacterial or viral infection in fish species such as Atlantic salmon. Using earlier results combined with integrated whole proteome and phosphoproteome data, we focused on the potential functions of these four proteins in subsequent experiments.

Motif-X analysis led to the identification of prospective novel kinases, including inactive serine/threonine-protein kinase VRK3-like, cyclin-G-associated kinase isoform X2, tyrosine-protein kinase HCK isoform X1, protein kinase C delta type-like and ribosomal protein S6 kinase alpha-3-like, which are well-known key modulators and initiators of the global phosphorylation response to *A*. *salmonicida* infection in Atlantic salmon.

Two proteins containing the common SxDx motif were identified. Vaccinia-related kinase 3 (VRK3), a member of the novel VRK family is located in the nucleus^31,32^. VRK3 displays alterations in the critical motifs essential for kinase activity, and includes two active vertebrate paralogs (VRK1 and VRK2), one or two orthologs in all metazoans, and an ortholog in most poxviruses, including the founding member, vaccinia virus B1R^31,33–37^. The VRK3 structure may be maintained to provide a binding surface for other proteins, a known function of many pseudokinases^38^. VRK3 also shows putative kinase activity in a cellular context^39^. The protein is highly expressed during development and promotes cell cycle progression by phosphorylating the nuclear envelope protein barrier to autointegration factor (BAF)^40^. Moreover, VRK3 suppresses extracellular signal regulated kinase (ERK) activity through direct binding to the MAP kinase phosphatase (MKP), vaccinia H1-related (VHR)^41^. In our experiments, phosphorylation of VRK3 at Ser118 and Ser151 was increased significantly in *A*. *salmonicida*-infected fish at 7 d. However, the underlying mechanisms and specific functions of these phosphorylation events are yet to be defined.

Cyclin G-associated kinase (GAK) is a protein containing highly conserved serine/threonine kinases with multiple functional domains^42^. GAK was initially reported to be associated with cyclin G and shown to play an important role in uncoating clathrin-coated vesicles (CCVs) in non-neuronal cells^43^. Another study demonstrated a role of GAK in clathrin-mediated endocytosis/vesicle trafficking in the cytoplasm and nucleus^44^. In addition, GAK has been implicated as an androgen receptor-interacting transcriptional coactivator^45^. GAK interacts specifically with interleukin 12 receptor β2 (IL-12Rβ2)^46^ that plays a central role in the initiation and control of cell-mediated immune responses to suppress IL-12-induced production of IFN-γ. Moreover, GAK is one of the two active kinases present in clathrin-coated vesicles and its Ser/Thr kinase activity is directed towards the l2 components of CCVs^47^. Based on integrated data from the current study and earlier experiments, we propose that GAK containing a phosphorylation site at Ser1049 has the potential to modulate immune responses in Atlantic salmon. The mechanisms linking GAK activity with immune pathways for development of a defense system against *A*. *salmonicida* in Atlantic salmon remain to be resolved.

Another protein, hematopoietic cell kinase (hck) containing the RxSx motif, is a member of the src family of tyrosine kinases and primarily expressed in myeloid cells. Expression of the gene is principally limited to cells of monocyte/macrophage and granulocyte lineages^48–51^. Levels of the gene increase with differentiation along either the macrophage or granulocyte pathways^52–54^ and are further augmented by activation stimuli, including LPS, in mature monocytes and macrophages^51,53,55^. A number of studies support a role of hck in the terminal differentiation of macrophages and/or signal transduction pathways of mature monocytes, macrophages lymphocytes, mast cells, and basophils^56–59^. We observed increased phosphorylation of this protein only at the Ser236 site with bacterial treatment.

The two other selected proteins had a common KxxS motif. The first was protein kinase C delta (PKCδ), which is involved in cytoskeletal regulation and apoptosis as a member of the PKC family^60,61^. Proteolytic activation of PKCδ is considered responsible for apoptotic execution^62,63^. Previous studies have focused on the mechanisms underlying PKCδ involvement in regulating cellular apoptosis and the cytoskeleton^60,63,64^. Notably, we observed significantly increased phosphorylation of this protein at the Ser297, Ser344 and Ser658 sites.

The second protein, ribosomal S6 protein kinase (RSK), phosphorylates serum response factors *in vitro* at an *in vivo* serum-stimulated phosphorylation site^65^. The protein is also regulated by extracellular signal regulated kinase (ERK) to inhibit the proapoptic Bcl2-family member, BCL2, an antagonist of cell death and caspase^66^. In this study, phosphorylation of RSK at Ser221 and Ser363 was increased significantly in infected fish at 7 d.

In conclusion, the five prospective novel kinases identified in our analyses (VRK3, GAK, HCK, PKCδ and RSK6) potentially contribute to resistance against bacterial propagation in fish. These proteins have common functions in regulation of apoptosis and the cytoskeleton in inflammatory and cellular pathways. However, further research is required to clarify whether these proteins participate in the defense mechanism against microbial pathogens and the underlying mechanisms.

Our findings, in conjunction with previous data, show that fga is the most central protein in the pathogenic network and may serve a potential biomarker of *A*. *salmonicida* infection in Atlantic salmon. In keeping with this proposal, earlier proteomic profiling studies support the utility of fga as a diagnostic marker of lung squamous cell carcinoma^67^. We therefore hypothesize the protein with well-established key functions than other proteins obtained from STRING-based functional network analysis in fish against bacteria.

In summary, we have presented an integrated analysis of the whole proteome and phosphoproteome showing that specific proteins are overexpressed and collectively drive the immune response against high-dose infection of *A*. *salmonicida* in kidneys of Atlantic salmon at 7 d. These data provide a framework for further investigation of Atlantic salmon resistance to *A*. *salmonicida*, and selection of indicators to improve resistance.

## Supplementary information

Supplemental Figures

Supplemental Table 1

Supplemental Table 2

Dataset 1

Dataset 2

Dataset 3

Dataset 4
