# Supplementary material for: Phosphoproteomic analyses of kidneys of Atlantic salmon infected with Aeromonas salmonicida
Source: Sci Rep. 2019 Feb 14;9:2101. doi: 10.1038/s41598-019-38890-3 (PMC6376026; doi:10.1038/s41598-019-38890-3)

**Phosphoproteomic analyses of kidneys of Atlantic salmon infected with *Aeromonas salmonicida***

Peng-fei Liu ^1, 2, 3^, Yishuai Du ^1, 2, 3^, Lingjie Meng ^1, 2, 3^, Xian Li ^1, 2, 3^, Dong Yang ^5, *^, Ying Liu ^4, *^

**1** Key Laboratory of Experimental Marine Biology, Institute of Oceanology, Chinese Academy of Sciences, Qingdao 266071, China
**2** Laboratory for Marine Fisheries Science and Food Production Processes, Qingdao National Laboratory for Marine Science and Technology, Qingdao 266235, China
**3** University of Chinese Academy of Sciences, Beijing 100039, China

**4** Dalian Ocean University, Dalian, PR China

**5** State Key Laboratory of Proteomics, Beijing Proteome Research Center, National Center for Protein Sciences (Beijing), Beijing Institute of Lifeomics; Beijing 102206, P. R. China.


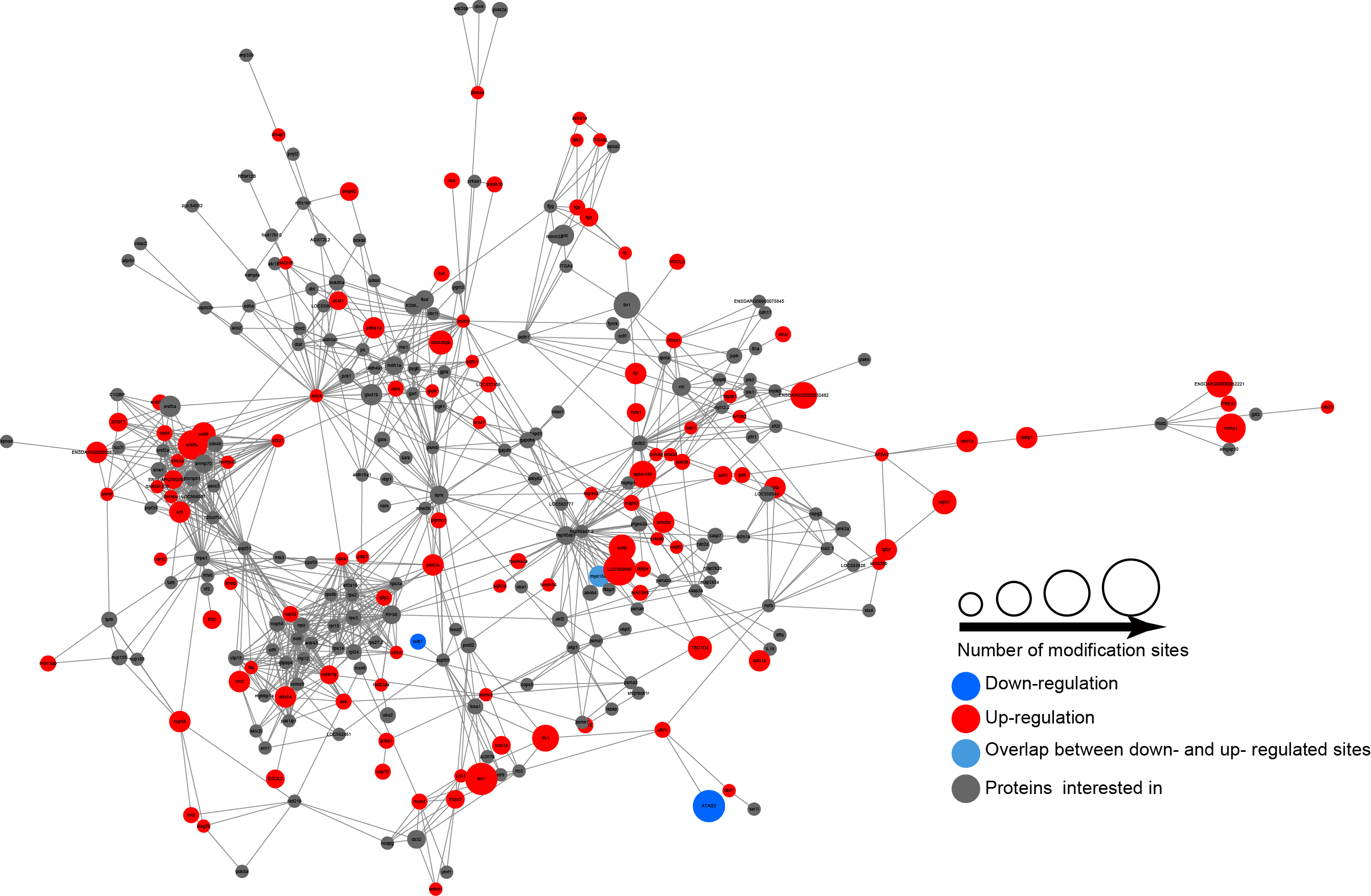

Supplement: Supplementary file 1 — Supplemental Figures [file 41598_2019_38890_MOESM1_ESM.doc]
